# Metabolic profiles and correlation with surgical outcomes in mesial versus neocortical temporal lobe epilepsy

**DOI:** 10.1111/cns.14209

**Published:** 2023-04-05

**Authors:** Hao‐yue Zhu, Yong‐xiang Tang, Ling Xiao, Shi‐rui Wen, Yuan‐xia Wu, Zhi‐quan Yang, Luo Zhou, Bo Xiao, Li Feng, Shuo Hu

**Affiliations:** ^1^ Department of Neurology Xiangya Hospital, Central South University Changsha Hunan 410008 China; ^2^ Department of Nuclear Medicine Xiangya Hospital, Central South University Changsha Hunan 410008 China; ^3^ Department of Neurosurgery Xiangya Hospital, Central South University 410008 Hunan Changsha China; ^4^ National Clinical Research Center for Geriatric Disorders Xiangya Hospital, Central South University Changsha Hunan 410008 China; ^5^ Department of Neurology Xiangya Hospital, Central South University (Jiangxi Branch) Nanchang Jiangxi 330000 China; ^6^ Key Laboratory of Biological Nanotechnology of National Health Commission Xiangya Hospital, Central South University Changsha Hunan 410008 China

**Keywords:** ^18^F‐FDG‐PET, metabolic profile, MTLE, NTLE, surgical prognosis

## Abstract

**Aims:**

Differentiating mesial temporal lobe epilepsy (MTLE) and neocortical temporal lobe epilepsy (NTLE) remains challenging. Our study characterized the metabolic profiles between MTLE and NTLE and their correlation with surgical prognosis using ^18^F‐FDG‐PET.

**Methods:**

A total of 137 patients with intractable temporal lobe epilepsy (TLE) and 40 age‐matched healthy controls were recruited. Patients were divided into the MTLE group (*N* = 91) and the NTLE group (*N* = 46). ^18^F‐FDG‐PET was used to measure the metabolism of regional cerebra, which was analyzed using statistical parametric mapping. The volume of abnormal metabolism in cerebral regions and their relationship with surgical prognosis were calculated for each surgical patient.

**Results:**

The cerebral hypometabolism of MTLE was limited to the ipsilateral temporal and insular lobes (*p* < 0.001, uncorrected). The NTLE patients showed hypometabolism in the ipsilateral temporal, frontal, and parietal lobes (*p* < 0.001, uncorrected). The MTLE patients showed extensive hypermetabolism in cerebral regions (*p* < 0.001, uncorrected). Hypermetabolism in NTLE was limited to the contralateral temporal lobe and cerebellum, ipsilateral frontal lobe, occipital lobe, and bilateral thalamus (*p* < 0.001, uncorrected). Among patients who underwent resection of epileptic lesions, 51 (67.1%) patients in the MTLE group and 10 (43.5%) in the NTLE group achieved Engel class IA outcome (*p* = 0.041). The volumes of metabolic increase for the frontal lobe or thalamus in the MTLE group were larger in non‐Engel class IA patients than Engel class IA patients (*p* < 0.05).

**Conclusions:**

The spatial metabolic profile discriminated NTLE from MTLE. Hypermetabolism of the thalamus and frontal lobe in MTLE may facilitate preoperative counseling and surgical planning.

## Graphical abstract

A striking difference in metabolic patterns using whole‐brain voxel‐based 18F‐FDG‐PET between mesial temporal lobe epilepsy (MTLE) and neocortical temporal lobe epilepsy (NTLE) was observed. The difference in metabolic patterns between the two temporal lobe epilepsy (TLE) subtypes may represent distinct epileptic networks that may guide the clinical classification of TLE. The presence of hypermetabolism involving the thalamus and frontal lobe in MTLE may be a predictor of an unfavorable seizure outcome in MTLE.

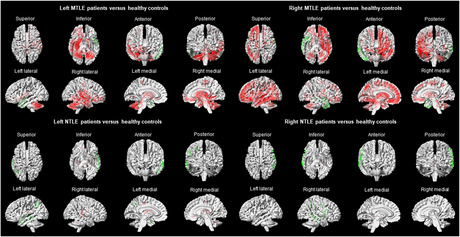

## 1 INTRODUCTION

Approximately 40% of patients with temporal lobe epilepsy (TLE) develop drug‐resistant epilepsy that may require surgical treatment.^1^ Classified by the epileptogenic area, two main syndromes have been described in TLE, mesial temporal lobe epilepsy (MTLE) and neocortical temporal lobe epilepsy (NTLE).^2^ A standardized anterior temporal lobectomy is the most targeted and efficient procedure for the treatment of MTLE,^3^ and tailored resection of the lateral temporal neocortex is often chosen for NTLE.^4^ However, the postoperative seizure outcome is not as favorable in NTLE as MTLE.^5^ Further improvements, such as precise identification of TLE subtypes and localization of the epileptogenic zone (EZ), should allow the elaboration of tailored surgical strategies for each patient to achieve better seizure outcomes.

Classification of the TLE subtype is based on seizure symptomatology, ictal and interictal scalp electroencephalography (EEG), and structural magnetic resonance imaging (MRI). However, most patients with MTLE or NTLE share similar clinical pictures, including viscerosensory aura, behavioral arrest, automatism, impaired consciousness, or secondary bilateral tonic–clonic seizures.^6^ They also exhibit similar patterns of EEG recordings that show episodic spikes and/or slow waves interictally localized over the unilateral temporal region.^4^, ^7^ For TLE with MRI‐invisible lesions, it would be much more difficult to differentiate NTLE from MTLE on the basis of structural brain imaging.^5^ The use of preoperative high‐resolution morphometric MRI or molecular imaging may help discriminate the subtypes of TLE patients and localize the region of seizure origination to improve surgical results via optimized presurgical planning.

Fluorine‐18 fluorodeoxyglucose positron emission tomography (^18^F‐FDG‐PET) is well accepted as an irreplaceable noninvasive presurgical molecular imaging method for patients with TLE.^8^ ^18^F‐FDG is a sensitive in vivo imaging tracer that has been widely used to reveal focal hypometabolic regions concordant with seizure onset.^9^ PET hypometabolism may be a promising indicator for presurgical evaluation of postoperative outcomes in drug‐resistant TLE patients.^10^ In‐depth evaluation combined with semiology, EEG findings, structural MRI, and PET would further improve EZ localization accuracy and postsurgical prediction in refractory epilepsy.^9^, ^11^, ^12^, ^13^ ^18^F‐FDG‐PET in neurodegenerative diseases with dementia reveals lobar‐specific patterns of hypometabolism related to particular types of dementia. For example, ^18^F‐FDG‐PET/computed tomography (CT) shows that hypometabolism in Alzheimer's disease first appears in the precuneus and posterior cingulate cortex and then in the temporoparietal lobes,^14^ and hypometabolism in the frontal and anterior temporal lobes with later involvement of the parietal lobes is more suggestive of frontotemporal dementia.^15^ We infer that MTLE and NTLE may also have different glucose metabolic features, which would assist in the classification of TLE subtypes and surgical evaluation of outcomes.

Our study retrospectively analyzed patients with TLE and reviewed their clinical, neuroelectrophysiological, neuroimaging, surgical, and follow‐up data. The patients were then categorized into MTLE and NTLE subgroups to screen interictal whole‐brain voxel‐based metabolic profiles using ^18^F‐FDG‐PET and statistical parametric mapping (SPM12) analyses. The correlation of different glucose metabolic levels was performed with surgical outcomes separately to investigate the underlying neural network and guide the planning of procedures.

## 2 METHODS

### 2.1 Study participants and clinical assessments

From January 2016 to June 2021, we recruited 137 patients with intractable TLE at Xiangya Hospital, Central South University. We collected the history, seizure symptoms, neurological examination, MRI, continuous noninvasive scalp video EEG, invasive stereo‐EEG (SEEG) recording, neuropsychological examination of all patients, surgery data, and outcomes of patients who underwent epilepsy surgery. The TLE patients were diagnosed based on the International League Against Epilepsy (ILAE) classification of epilepsy types.^16^, ^17^ Clinical data of all patients were complete, and the number of episodes was greater than two times per year. The lateralization and localization of the EZ and therapy were determined using a multidisciplinary team (MDT) consisting of two experienced epileptologists, a neuroelectrophysiologist, a neuroradiologist, aneuropsychologist, and two neurosurgeons. According to these comprehensive data and the current categories proposed in previous studies,^18^, ^19^ the following inclusion criteria were used for patients with MTLE: (a) unilateral mesial temporal lobe seizure onset using continuous noninvasive scalp video EEG or SEEG recordings; (b) MRI evidence of pathology located within the epileptogenic mesial temporal lobe; and (c) concordant PET finding of hypometabolism in the interictal temporal lobe. Patients must meet item (a) and one or both items (b) and (c) to be enrolled in the MTLE group. The following inclusion criteria were used for patients with NTLE: (a) the presence of a structural abnormality in the unilateral neocortical temporal lobe on MRI and a compatible ictal onset was identified during continuous scalp video EEG monitoring or (b) the structural MRI was normal, but SEEG confirmed neocortical ictal onset. Patients with a history of severe systemic or psychiatric illness, drug abuse, and substance use disorder were excluded. The prognostication of the outcome was graded according to the Engel Surgical Outcome scale.^20^ Forty healthy controls (HCs), matched for age and sex, were recruited randomly in our study. ^18^F‐FDG‐PET was used to measure regional cerebral metabolism in all participants. The Institutional Review Committee of Xiangya Hospital approved the protocol before patient recruitment commenced. All participants provided informed consent in writing before participation. For participants under the age of 16 years, consent was obtained from their legal guardians.

### 2.2 ^18^F‐FDG‐PET image examination

Within 1 week of clinical evaluation, ^18^F‐FDG‐PET scanning was performed using a Discovery Elite PET/CT scanner (GE Healthcare). Antiseizure medications (ASMs) were discontinued for at least 24 h, and patients fasted for at least 6 h before ^18^F‐FDG injection. Under strict surveillance, patients had no clinically visible seizures within 24 h and had a continuous EEG recording 2 h before tracer injection to ensure that ^18^F‐FDG was not implemented in a postictal situation.^21^ ^18^F‐FDG was injected intravenously through the cubital vein at a dose of 3.7 MBq/kg over 1 min. Static PET/CT images were acquired in three dimensions over 60 min after tracer injection. Participants were laid flat in the PET scanner, which allowed slices to be parallel to the canthomeatal line. The full width of the scan at half maximum was 5.4 mm. The detailed protocol of the PET scan has been described previously.^22^

### 2.3 Data analyses

SPM12 (Wellcome Department of Cognitive Neurology, UK) implemented in MATLAB was used to perform image processing. The spatial standard of individual ^18^F‐FDG‐PET image volumes was normalized into Montreal Neurological Institute (MNI) space with 2 × 2 × 2 voxel sizes. We used an 8‐mm full‐width‐half‐maximum Gaussian kernel to blur individual variations in the rotative anatomy and improve within‐participant spatial alignment, which smoothed the data for statistical analysis. Because different laterality of seizure onset may confuse the data, we compared the cerebral metabolism of patients with MTLE on the left and right side, and patients with NTLE on the left and right side separately against HCs. A general linear model was used to administer voxel‐by‐voxel univariate statistical tests.

Metabolic alterations were obtained at an uncorrected height threshold (significance of voxel level) of *p* < 0.001, with cluster size (K_E_) above 20 contiguous voxels. After SPM12 preprocessing, xjView toolkits (http://www.alivelearn.net/xjview) were used to visualize, report, and label the significant clusters anatomically. Data on metabolic pattern information about the clusters were obtained, including the number of voxels, peak intensity of each cluster, and anatomical location, which used automated anatomical labeling (AAL) to approximate Brodmann areas. The volume of metabolism changes in the temporal lobe, frontal lobe, parietal lobe, occipital lobe, insula lobe, limbic system, basal ganglia thalamus, brainstem, and cerebellum for each surgical patient was calculated separately using the xjView toolkit.

### 2.4 Statistical analyses

Statistical analyses were performed using SPSS software for Windows (IBM SPSS Statistics, Version 26.0, IBM Corp). We used Shapiro–Wilk tests to analyze the normality of all quantitative variables, which are summarized as the means ± standard deviation (SD) or medians (interquartile range, IQR). The age of the participants showed a normal distribution. The Mann–Whitney U test or Student's *t*‐test was used for comparisons of quantitative variables between the MTLE and NTLE groups, all patients, and HCs. The chi‐squared test or Fisher's test was used for qualitative variables. All statistical tests were two‐sided, and *p* < 0.05 indicated statistical significance.

For PET image analysis, analysis of covariance (ANCOVA) was used to compare baseline glucose uptake values of each epilepsy group (left and right MTLE, then left and right NTLE) and HCs, with the group as the between‐subject factor and age and sex as confounding covariates. A two‐sample *t*‐test was used to compare the different groups. Metabolic changes in the whole brain and cerebellum were calculated, and the MTLE and NTLE groups were compared to HCs separately. The volumes of metabolic changes, including hypermetabolism and hypometabolism, in temporal and extratemporal areas and their relationship with surgical prognosis for each surgical patient were also calculated. The Mann–Whitney U test was used for the comparison of the volumes between different groups.

## 3 RESULTS

### 3.1 Demographic and clinical characteristics

There were 91 MTLE (56 men and 35 women; mean age 23.1 ± 7.3 years) and 46 NTLE patients (28 men and 18 women; mean age 19.8 ± 10.8 years) included in this study. The mean age for all patients was 23.0 ± 8.8 years. Forty volunteers (21 men and 19 women; mean age 21.6 ± 8.4 years) were recruited as HCs. For the clinical characteristics, there were no significant differences in onset age, duration of epilepsy, frequency of seizures, or lateralization distribution between the two subgroups. Twelve (13.2%) patients in the MTLE group and 13 (28.3%) patients in the NTLE group underwent SEEG monitoring. The demographic and clinical characteristics of the participants are summarized in Table 1.

**TABLE 1:** Demographics of participants and clinical characteristics of patients.

	MTLE (*N* = 91)	NTLE (*N* = 46)	*p* value	Total patients (*N* = 137)	HC (*N* = 40)	*p* value
Age (mean ± SD)	23.1 ± 7.3	19.8 ± 10.8	0.065^a^	23.0 ± 8.8	21.6 ± 8.4	0.802^a^
Sex						
Male, *n* (%)	56 (61.5%)	28 (60.9%)	0.939^b^	84 (61.3%)	21 (52.5%)	0.318^b^
Female, *n* (%)	35 (38.5%)	18 (39.1)		53 (38.7)	19 (47.5%)	
Onset age (year), median (IQR)	13 (9–20)	11 (5–16)	0.142^c^	13 (7–19)	—	NA
Duration of epilepsy (year), median (IQR)	8 (4–15)	6 (3–11)	0.139^c^	8 (4–13)	—	NA
Frequency of seizure, *n* (%)						
< Once half a year	6 (6.6%)	2 (4.4%)	0.207^c^	8 (5.8%)	—	NA
< Once a month	8 (8.8%)	7 (15.2%)		15 (10.9%)	—	NA
≥ Once a month	57 (62.6%)	19 (41.3%)		76 (55.5%)	—	NA
Daily	20 (22.0%)	18 (39.1%)		38 (27.7%)	—	NA
SEEG, *n* (%)	12 (13.2%)	13 (28.3%)	0.031^b^ ^,^ ^d^	25 (18.2%)	—	NA
lateralization, *n* (%)						
Left	48 (53.3%)	28 (60.9%)	0.402^b^	76 (55.5%)	—	NA
Right	43 (46.7%)	18 (39.1%)		61 (44.5%)	—	NA

Abbreviations: HC, heathy controls; MTLE, mesial temporal lobe epilepsy; NA, not applicable; NTLE, neocortical temporal lobe epilepsy; SD, standard deviation; SEEG, stereoelectroencephalography.

^a^ Student's *t*‐test.

^b^ Chi‐square test or Fisher exact test.

^c^ Mann–Whitney *U* test.

^d^ Values indicate significant difference (*p* < 0.05).

### 3.2 Metabolic abnormalities in patients with MTLE

**FIGURE 1:**
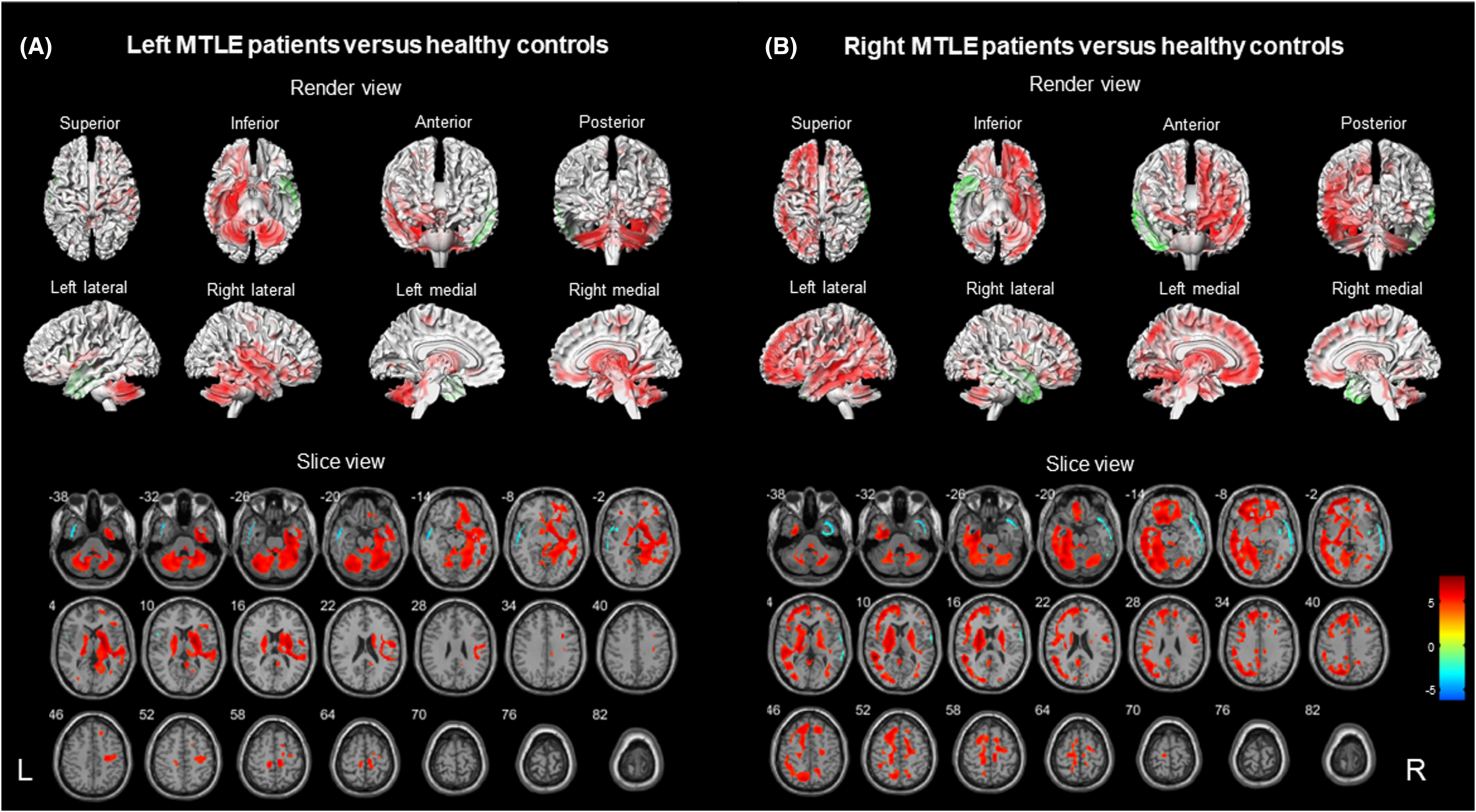
Comparison of patients with different MTLE (*N* = 91) versus healthy controls (*N* = 40) (*p* < 0.001, uncorrected). Voxels with significantly low uptake are shown in green, and voxels with high uptake are shown in red. (A) Left MTLE patients (*N* = 48) versus healthy controls were performed in render view (upper) and slice view (below). (B) Right MTLE patients (*N* = 43) versus healthy controls were performed in render view (upper) and slice view (below). MTLE, mesial temporal lobe epilepsy.

The epileptogenic regions in the MTLE group were well lateralized to the left MTLE (*n* = 46) and right MTLE (*n* = 43) based on routine clinical reports with concordant static ^18^F‐FDG‐PET hypometabolism. Comparison of MTLE to HCs revealed that the cerebral hypometabolism of MTLE was limited to the ipsilateral temporal and insular lobe (*p* < 0.001, uncorrected). Extensive changes in cerebral hypermetabolism were present in the contralateral temporal, bilateral frontoparietal, occipital regions, limbic lobe, corpus callosum, thalamus, basal ganglia, brainstem, and cerebellum (*p* < 0.001, uncorrected). The affected limbic lobe included the cingulate gyrus, parahippocampal gyrus, and amygdala. The render and slice view of MTLE compared to HC is shown in Figure 1. Table S1 lists the peaks of the most significant voxels and shows the location of the voxels within each cluster.

### 3.3 Metabolic abnormalities in patients with NTLE

**FIGURE 2:**
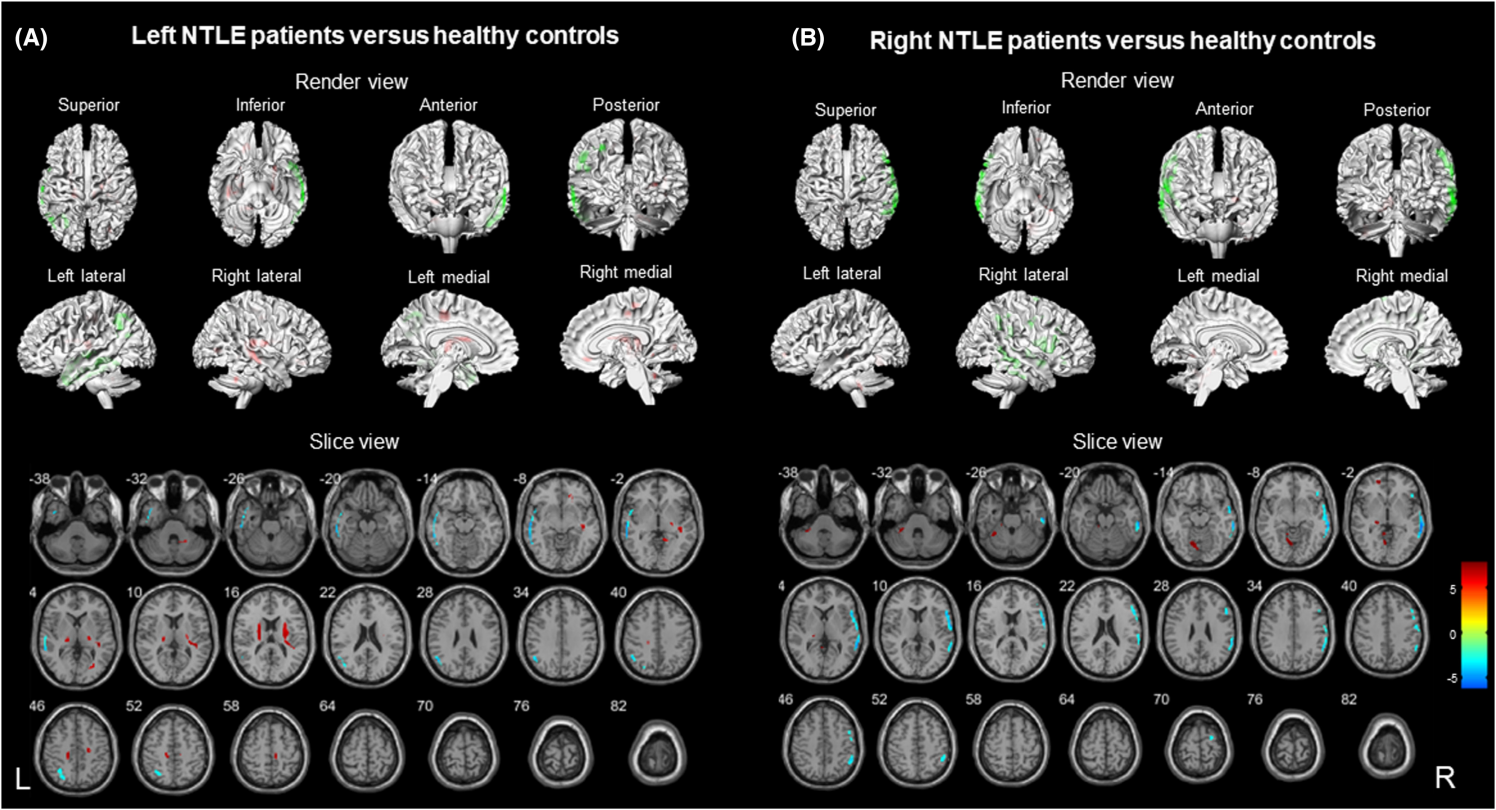
Comparison of patients with different NTLE (*N* = 46) versus healthy controls (*N* = 40) (*p* < 0.001, uncorrected). Voxels with significantly low uptake are shown in green, and voxels with high uptake are shown in red. (A) Left NTLE patients (*N* = 28) versus healthy controls were performed in render view (upper) and slice view (below). (B) Right NTLE patients (*N* = 18) versus healthy controls were performed in render view (upper) and slice view (below). NTLE, neocortical temporal lobe epilepsy.

The epileptogenic regions of the NTLE group were also well lateralized to the left NTLE (*n* = 28) and right NTLE (*n* = 18). In addition to the ipsilateral temporal lobe, patients with NTLE showed hypometabolism in the ipsilateral frontal lobe and parietal lobe (*p* < 0.001, uncorrected). Cerebral hypermetabolism in patients with NTLE was limited to the contralateral temporal lobe and cerebellum, ipsilateral frontal lobe, occipital lobe, insula, and bilateral thalamus (*p* < 0.001, uncorrected). The render and slice views of NTLE compared to HC are shown in Figure 2. Notably, unlike MTLE, hypermetabolism was observed only in the contralateral cerebellum.

### 3.4 The volume of hypermetabolism in temporal and extratemporal regions

**FIGURE 3:**
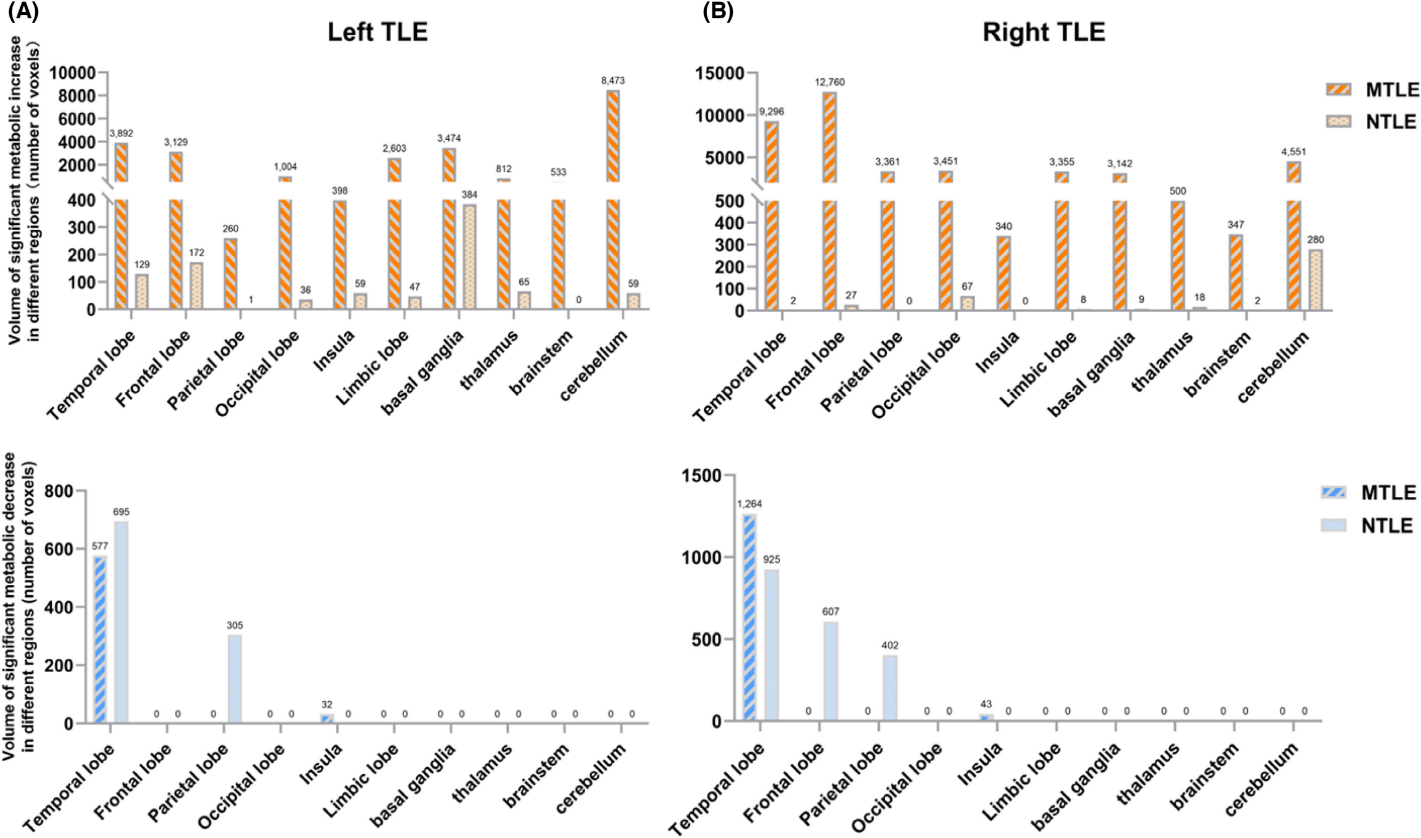
Involvement volume of abnormal metabolism in patients with MTLE compared to patients with NTLE. We compared the metabolic increased (upper, orange) and decreased (below, blue) volumes of involvement of each cerebral region in the two groups. In the left (A) and right (B) TLE, MTLE patients had significantly higher hypermetabolic volumes in each of these regions than NTLE patients, including the contralateral temporal lobe and frontal, parietal, occipital lobe, insula, limbic lobe, basal ganglia, thalamus, brainstem, and cerebellum. The difference between the two types of right TLEs was greater than the difference between the left TLEs. The hypometabolic region of MTLE involved only the temporal lobe and insula. The hypometabolic region of left NTLE involved the temporal and parietal lobes, and the hypometabolic region of right NTLE involved the temporal, parietal, and frontal lobes. MTLE, mesial temporal lobe epilepsy; NTLE, neocortical temporal lobe epilepsy; TLE, temporal lobe epilepsy.

Significant differences were found in the metabolic patterns of MTLE and NTLE, especially in the hypermetabolic regions. Figure 3 demonstrates the involved volume of metabolic abnormalities in patients with MTLE compared to patients with NTLE. MTLE patients had significantly higher hypermetabolic volumes in each of the regions than NTLE patients, including the contralateral temporal lobe and frontal, parietal, occipital lobe, insula, limbic lobe, basal ganglia, thalamus, brainstem, and cerebellum. The difference in hypermetabolism between the two types of right TLEs was greater than the differences between the left TLEs. The hypometabolic region of MTLE involved only the temporal lobe and insula, and the hypometabolic regions of left NTLE involved the temporal and parietal lobes, and right NTLE involved the temporal, parietal, and frontal lobes.

### 3.5 The correlation between surgical outcome and abnormal metabolic volume of specific extratemporal brain regions

Based on available clinical data, including detailed neurological history, physical examination, MRI, video EEG or SEEG, and joint decision of MDT, 76 MTLE patients (83.5%) and 23 NTLE patients (50%) underwent epilepsy surgery. Seventy‐five patients with MTLE underwent anterior temporal lobectomy, and the remaining patient with MTLE underwent surgery with target destruction. All patients with NTLE underwent tailored resection of the lateral temporal neocortex. Except for one patient who underwent target destruction, postoperative pathology was performed in 98 patients (Table 2). With an average follow‐up of 2.9 years after surgery, 61 of 99 patients achieved Engel class IA outcomes. Seizure‐free status was more frequently achieved in MTLE patients than NTLE patients (67.1% vs. 43.5%, *p* = 0.041). We divided the two groups into Engel class IA, which represented good surgical outcomes, and non‐Engel class IA for volume comparison. The hypermetabolic volumes for the frontal lobe and thalamus in the MTLE group were larger in non‐Engel class IA patients than Engel class IA patients (*p* < 0.05), but no other significantly different hyper‐ and hypometabolic regions were found between the non‐Engel class IA and Engel class IA patients. There was no significant difference between the hyper and hypometabolic regions in the NTLE patients with different surgical outcomes (Table 3).

**TABLE 2:** Surgical data of patients who underwent surgery.

	MTLE (*n* = 76)	NTLE (*n* = 23)	*p* value
Surgical approach, *n* (%)			
Anterior temporal lobectomy	75 (98.7%)	0 (0.0%)	NA
Tailored resection of the lateral temporal neocortex	0 (0.0%)	23 (100.0%)	
Target destruction	1 (1.3%)	0 (0.0%)	
Postoperative pathology			
HS, *n* (%)	33 (44.0%)	0 (0.0%)	NA
FCD, *n* (%)	11 (14.7%)	7 (30.4%)	
Gliosis, *n* (%)	25 (33.3%)	6 (26.1%)	
Tumor, *n* (%)	2 (2.7%)	3 (13.0%)	
Other, *n* (%)	4 (5.3%)	7 (30.4%)	
Surgical outcomes			
Engel class IA, *n* (%)	51 (67.1)	10 (43.5%)	0.041^a^ ^,^ ^b^
Non‐Engel class IA, *n* (%)	25 (32.9%)	13 (56.5%)	

Abbreviations: FCD, focal cortical dysplasia; HS, hippocampal sclerosis; MTLE, mesial temporal lobe epilepsy; NA, not applicable; NTLE, neocortical temporal lobe epilepsy.

^a^ Chi‐square test or Fisher Exact test.

^b^ Values indicate significant difference (*p* < 0.05).

**TABLE 3:** Significant change in glucose metabolism involving different cerebral regions in patients with MTLE or NTLE with different surgical outcomes.

	MTLE (*n* = 76)		*p* value	NTLE (*n* = 23)		*p* value
	Engel class IA *n* = 51	Non‐Engel class IA *n* = 25		Engel class IA *n* = 10	Non‐Engel class IA *n* = 13	
The volume of metabolic increase (number of voxels), median (IQR)						
Temporal lobe	4 (0–105)	40 (0–190)	0.351^a^	0 (0–125)	0 (0–62.5)	0.597^a^
Frontal lobe	30 (0–128)	73 (14.5–331)	0.027^a^ ^,^ ^b^	25 (0–955)	0 (0–315)	0.493^a^
Parietal lobe	1 (0–146)	66 (0–222)	0.107^a^	0 (0–317.75)	0 (0–49.5)	0.805^a^
Occipital lobe	0 (0–40)	0(0–46.5)	0.466^a^	0 (0–131.5)	0 (0–70.5)	0.827^a^
Insula	0 (0–6)	0 (0–22)	0.324^a^	0 (0–7.5)	0 (0–13)	0.970^a^
Limbic system	0 (0–37)	16 (0–85.5)	0.254^a^	6 (0–112)	0 (0–172.5)	0.632^a^
Basal ganglia	0 (0–23)	5 (0–81)	0.199^a^	0 (0–30.5)	0 (0–23.5)	0.970^a^
Thalamus	0 (0–0)	0 (0–35)	0.019^a^ ^,^ ^b^	0 (0–0)	0 (0–0)	0.791^a^
Brainstem	0 (0–0)	0 (0–0)	0.177^a^	0 (0–0)	0 (0–0)	0.177^a^
Cerebellum	0 (0–31)	0 (0–81)	0.756^a^	0 (0–107.25)	0 (0–39.5)	0.610^a^
The volume of metabolic decrease (number of voxels), median (IQR)						
Temporal lobe	22 (0–163)	54 (0–597.5)	0.273^a^	186 (0–651)	18 (0–583)	0.660^a^
Frontal lobe	0 (0–24)	0 (0–55)	0.876^a^	192.5 (0–519)	0 (0–150.5)	0.113^a^
Parietal lobe	0 (0–23)	0 (0–10.5)	0.902^a^	10.5 (0–833.5)	42 (0–125.5)	0.942^a^
Occipital lobe	0 (0–0)	0 (0–0)	0.237^a^	0 (0–670)	0 (0–22)	0.942^a^
Insula	0 (0–0)	0 (0–0)	0.072^a^	0 (0–36)	0 (0–0)	0.335^a^
Limbic system	0 (0–0)	0 (0–0)	0.197^a^	0 (0–292)	0 (0–7.5)	0.286^a^
Basal ganglia	0 (0–0)	0 (0–0)	0.590^a^	0 (0–8)	0 (0–1.5)	0.630^a^
Thalamus	NA	NA	NA	NA	NA	NA
Brainstem	0 (0–0)	0 (0–0)	0.484^a^	NA	NA	NA
Cerebellum	0 (0–0)	0 (0–0)	0.493^a^	0 (0–69.75)	0 (0–0)	0.323^a^

Abbreviations: MTLE, mesial temporal lobe epilepsy; NA not applicable; NTLE, neocortical temporal lobe epilepsy.

^a^ Mann–Whitney *U* test.

^b^ Values indicate significant difference (*p* < 0.05).

## 4 DISCUSSION

Our study used whole‐brain voxel‐based ^18^F‐FDG‐PET and found a striking difference in metabolic patterns between MTLE and NTLE. Patients with MTLE exhibited a characteristic ipsilateral hypometabolism in the temporal lobe and bilateral cortical–subcortical hypermetabolism pattern. In contrast, NTLE patients showed more extensive hypometabolic regions, including the temporal and front‐parietal cortex, and hypermetabolism in certain cortical regions. The difference in metabolic patterns between the two TLE subtypes may represent distinct epileptic networks that may guide the clinical classification of epilepsy. Hypermetabolism involving the thalamus and frontal lobe may be a predictor of an unfavorable seizure outcome in TLE, which facilitated preoperative counseling and surgical planning.

^18^F‐FDG‐PET has been widely used in patients with medically refractory focal epilepsy for the detection of epileptogenic lesions.^9^, ^13^ Pathological reduction in glucose metabolism indicates focal neuronal and synaptic loss associated with epileptogenic brain regions.^23^ Consistent with Guedj et al.^10^ our study identified two patterns of cerebral hypometabolism in the MTLE and NTLE groups, which were mostly related to EZs. Glucose hypometabolism was present in the ipsilateral hippocampus and insular lobe in MTLE. The medial structure of the temporal lobe, including the hippocampus, is the seizure origination location in MTLE, and the cortical insula commonly becomes the earliest involved structure as spikes propagate via the Papez circuit. Recurrent spike distribution and seizure propagation from the mesial temporal lobe to the insular cortex contribute to focal metabolic impairment in the insula or lead to insular atrophy.^24^, ^25^ The hypometabolic region in NTLE is primarily located in the temporal neocortex associated with the EZ. Notably, we also found some restricted extratemporal cortical regions in the frontal and parietal lobes showing decreased glucose metabolism in NTLE patients, which suggested more extensive brain regions and connected propagated networks affected in NTLE. Therefore, it is plausible that patients with NTLE generally demonstrate behavioral arrest with awareness impairment at the early stage^26^, ^27^ followed by motor signs as the seizure activity spreads to the frontoparietal convexity.^28^, ^29^

Notably, increasing evidence suggests that epilepsy is a network disorder, and mixed cerebral metabolic patterns were found recently.^30^ Both hypermetabolism and hypometabolism may have pathophysiological significance in the epileptic network and contribute to seizure origination or propagation. Our study found a more significant disparity in hypermetabolic patterns between MTLE and NTLE patients. They both showed hypermetabolism in cortical and subcortical regions but with different locations. Glucose metabolism is commonly enhanced in the bilateral basal ganglia, brainstem, thalamus, corpus callosum, cingulate gyrus, and frontoparietal‐occipital cortex, which suggests that the propagation pathway in these structures is likely involved in the MTLE group.^31^, ^32^, ^33^, ^34^ The thalamus and brainstem are distinctly connected with the cingulate gyrus and cerebral cortex.^35^, ^36^ A focal seizure that originates from the mesial structure of the temporal lobe generally spreads to key subcortical regions, such as the brainstem and bilateral thalamus, which result in widespread metabolic disturbance in midline subcortical structures and neocortex.^37^, ^38^ Large areas of hypermetabolism in contralateral cerebral regions of MTLE seem to result from the restoration of chemical homeostasis.^39^

Although cortical and subcortical hypermetabolism areas beyond the EZs are also present in patients with NTLE, it is much more restricted in the range of glucose metabolism. Notably, the key hypermetabolic region of NTLE is typically the bilateral thalamus, which suggests an underlying propagation pathway from the lateral neocortex of the temporal lobe through the basal ganglia to the contralateral hemisphere that contributes to bilateral tonic–clonic seizures.^40^ Considering the extent of involvement, we speculate that in addition to the trans‐thalamic circuits, other propagating pathways may exist in NTLE.^41^

We also observed significant hypermetabolism in the cerebellum, which is consistent with previous functional MRI and SPECT studies.^42^, ^43^ Sufficient data indicate that the cerebellum is engaged during seizures, which manifests as reduced gray matter volumes,^44^ increased cerebellar blood flow, and neuronal activities.^45^, ^46^ Increased interictal metabolic activities in the cerebellum have also been reported in animal models of epilepsy.^47^ The cerebellar metabolic changes in TLE patients in our study suggest a functional significance of temporal‐cerebellar connections and the potential ability for cerebellar activation to inhibit seizures.^48^ Another possibility is that abnormal glucose metabolism in the cerebellum may be compensatory as one of the downstream targets via divergent output pathways from the temporal lobe.^46^ Notably, the bilateral cerebellum is likely affected in MTLE, and abnormal metabolism is only found in the contralateral cerebellar hemisphere in the NTLE group, which highlights the potential importance of the cerebellum in the differentiation of epilepsy phenotypes in TLE.

We further assessed metabolic features and surgical outcomes in patients with two subtypes of TLE. We found that patients with MTLE had a worse surgical prognosis when thalamic or frontal hypermetabolism was present. Lesional mesial temporal lobe epilepsy generally includes hippocampal sclerosis, focal cortical dysplasia, or local neurodevelopmental tumors.^49^ Due to their limited focal damage, standard anterior temporal lobectomy offers comparatively favorable outcomes (50%–80% seizure‐free rate).^50^ However, if combined with an extended frontal or thalamic ^18^F‐FDG metabolic disturbance, actual lesions associated with epileptogenesis or seizure‐related networks in MTLE may be larger than they appear on structural imaging. Therefore, the removal of only the anterior temporal lobe region may not be sufficient for postsurgical seizure control. Surgical procedure selection should be comprehensively determined if MTLE patients show thalamic or frontal involvement on ^18^F‐FDG‐PET images. Precise and sufficient location of epileptogenic foci, including invasive EEG, should be carefully performed for better surgical outcomes

Several potential limitations in our study should be mentioned. First, this study was a retrospective study, which inevitably brings selection biases. Second, the sample size is relatively small, especially for the NTLE group, which only accounts for 10% of TLE,^51^ which may limit the statistical power of investigating abnormal metabolic patterns in patients. Third, due to the limitation of “real‐world” clinical studies, not all patients recruited in our study underwent SEEG monitoring, which is considered the gold standard for identifying seizure origination. However, our results lay the groundwork for important future studies, such as assisting TLE subtype classification and surgical prognosis assessment. Although ASMs were discontinued for at least 24 h before the PET scan, they may still have residual effects on brain metabolism. However, most PET studies in epilepsy shared this limitation, which is hard to overcome.

## 5 CONCLUSION

Distinctive characteristic features of ^18^F‐FDG‐PET metabolic profiles have been identified in NTLE and MTLE patients. Glucose hypometabolism was associated with the EZ, and the hypermetabolic regions suggest their different propagation networks and underlying compensatory mechanisms. Notably, the presence of hypermetabolism involving the thalamus and frontal lobe in MTLE may be a predictor of larger epileptogenic foci and an unfavorable seizure surgical outcome. Comprehensive evaluations, including invasive EEG, should be applied in clinical practice.

## AUTHOR CONTRIBUTIONS

Shuo Hu, Li Feng, Hao‐yue Zhu, and Yong‐xiang Tang designed the study. Hao‐yue Zhu and Yong‐xiang Tang analyzed the data. Shuo Hu and Li Feng supervised the study. Hao‐yue Zhu, Li Feng, and Yong‐xiang Tang wrote the manuscript. All authors interpreted the data and revised the manuscript.

## FUNDING INFORMATION

This work was supported by the following funding source: National Key Research and Development Program of China (No. 2022YFC2503804), National Natural Science Foundation of China (No. 91859207, 81771873, 82272045, 82071461, 82271503, and 81801740), Key Program of Ministry of Industry and Information Technology of China (No. CEIEC‐2022‐ZM02‐0219), Science and Technology Innovation Team Talent Project of Hunan Province (No. 2021RC4056), Natural Science Foundation of Hunan Province (No. 2021JJ31060 and 2020JJ5922), Clinical Research Foundation of the National Clinical Research Center for Geriatric Diseases (XIANGYA)(No. 2020LNJJ01), China Postdoctoral Science Foundation (No. 2022M723561), and Innovative Construction Foundation of Hunan Province (No. 2021SK4001).

## CONFLICT OF INTEREST STATEMENT

All authors declare no conflicts of interest in this work.

## Supporting information


Table S1.

## Data Availability

Anonymized data not published within this article will be made available by request from any qualified investigator.
