# Supplementary material for: Metabolic profiles and correlation with surgical outcomes in mesial versus neocortical temporal lobe epilepsy
Source: CNS Neurosci Ther. 2023 Apr 5;29(9):2656–65. doi: 10.1111/cns.14209 (PMC10401181; doi:10.1111/cns.14209)
Supplement: Supplementary file 1 — Table S1. [file CNS-29-2656-s001.docx]

**TABLE S1.** Location and peaks of significant reduction/increasing in glucose metabolism in patients with MTLE or NTLE compared with heathy controls.

| Region | Brodmann Area | Coordinates(mm) | | | Peak level | | | | | Cluster level | | | |
| --- | --- | --- | --- | --- | --- | --- | --- | --- | --- | --- | --- | --- | --- |
|  |  | X | Y | Z | p(FWE-corr) | p(FDR-corr) | T | Z | p(unc) |  | p(FWE-corr) | K_E_ | p(unc) |
| Left MTLE vs Control | | | | | | | | | | | | | |
| Hippocampus_R (aal3v1) |  | 32 | -8 | -24 | 0.000 | 0.000 | 6.224 | 5.639 | 0.000 |  | 0.000 | 25001 | 0.000 |
| Fusiform_R (aal3v1) |  | 42 | -50 | -24 | 0.000 | 0.000 | 6.169 | 5.598 | 0.000 |  |  |  |  |
| Frontal_Inf_Tri_R (aal3v1) |  | 38 | -32 | 8 | 0.001 | 0.000 | 5.770 | 5.290 | 0.000 |  |  |  |  |
| Paracentral_Lobule_R (aal3v1) |  | 8 | -26 | 60 | 0.011 | 0.000 | 5.141 | 4.787 | 0.000 |  | 0.136 | 249 | 0.039 |
| Paracentral_Lobule_L (aal3v1) | 6 | -10 | -32 | 60 | 0.101 | 0.001 | 4.475 | 4.231 | 0.000 |  | 0.223 | 189 | 0.068 |
| Left Frontal Lobe Sub-Gyral |  | -18 | -24 | 52 | 0.856 | 0.003 | 3.462 | 3.340 | 0.000 |  |  |  |  |
| Frontal_Inf_Oper_R (aal3v1) |  | 30 | 8 | 34 | 0.187 | 0.001 | 4.262 | 4.048 | 0.000 |  | 0.601 | 71 | 0.246 |
| Left Middle Frontal Gyrus |  | -32 | 38 | -2 | 0.311 | 0.001 | 4.067 | 3.879 | 0.000 |  | 0.620 | 67 | 0.259 |
| Cingulate_Mid_R (aal3v1) |  | 10 | -24 | 32 | 0.483 | 0.001 | 3.868 | 3.703 | 0.000 |  | 0.806 | 32 | 0.438 |
| Occipital_Mid_L (aal3v1) |  | -30 | -76 | 4 | 0.636 | 0.002 | 3.713 | 3.566 | 0.000 |  | 0.865 | 21 | 0.537 |
| Supp_Motor_Area_R (aal3v1) | 6 | 14 | 8 | 56 | 0.676 | 0.002 | 3.672 | 3.529 | 0.000 |  | 0.745 | 43 | 0.366 |
| Frontal_Sup_2_R (aal3v1) |  | 16 | -10 | 60 | 0.693 | 0.002 | 3.654 | 3.513 | 0.000 |  | 0.740 | 44 | 0.361 |
| Frontal_Sup_2_R (aal3v1) |  | 14 | 32 | 46 | 0.745 | 0.002 | 3.599 | 3.463 | 0.000 |  | 0.762 | 40 | 0.384 |
| Lingual_L (aal3v1) | 18 | -14 | -78 | 0 | 0.750 | 0.002 | 3.593 | 3.458 | 0.000 |  | 0.681 | 55 | 0.306 |
| SupraMarginal_R (aal3v1) |  | 52 | -26 | 24 | 0.810 | 0.003 | 3.523 | 3.395 | 0.000 |  | 0.871 | 20 | 0.547 |
| Temporal_Pole_Mid_L (aal3v1) |  | -48 | 10 | -28 | 0.000 | 0.001 | -6.319 | 5.711 | 0.000 |  | 0.010 | 616 | 0.003 |
| Temporal_Mid_L (aal3v1) |  | -56 | -2 | -20 | 0.002 | 0.002 | -5.569 | 5.132 | 0.000 |  |  |  |  |
| Temporal_Mid_L (aal3v1) |  | -60 | -8 | -12 | 0.003 | 0.002 | -5.480 | 5.061 | 0.000 |  |  |  |  |
| Insula_L (aal3v1) |  | -40 | 4 | -2 | 0.691 | 0.089 | -3.656 | 3.515 | 0.000 |  | 0.806 | 32 | 0.438 |
| Right MTLE vs Control | | | | | | | | | | | | | |
| Temporal_Sup_L (aal3v1) |  | -52 | -42 | 12 | 0.000 | 0.000 | 6.629 | 5.908 | 0.000 |  | 0.000 | 36583 | 0.000 |
| ParaHippocampal_L (aal3v1) | 36 | -28 | -14 | -28 | 0.000 | 0.000 | 6.161 | 5.562 | 0.000 |  |  |  |  |
| Frontal_Inf_Orb_2_L (aal3v1) |  | -40 | 36 | -8 | 0.000 | 0.000 | 6.062 | 5.488 | 0.000 |  |  |  |  |
| Supp_Motor_Area_R (aal3v1) |  | 8 | -24 | 58 | 0.015 | 0.000 | 5.115 | 4.747 | 0.000 |  | 0.001 | 952 | 0.000 |
| Right Medial Frontal Gyrus |  | 18 | -10 | 54 | 0.022 | 0.000 | 4.988 | 4.644 | 0.000 |  |  |  |  |
| Supp_Motor_Area_R (aal3v1) | 6 | 12 | 8 | 54 | 0.187 | 0.000 | 4.305 | 4.073 | 0.000 |  |  |  |  |
| Right Precentral Gyrus | 6 | 50 | -4 | 22 | 0.041 | 0.000 | 4.804 | 4.493 | 0.000 |  | 0.054 | 355 | 0.014 |
| Postcentral_R (aal3v1) |  | 50 | -12 | 28 | 0.148 | 0.000 | 4.387 | 4.143 | 0.000 |  |  |  |  |
| Right Frontal Lobe/Sub-Gyral |  | 34 | 0 | 24 | 0.640 | 0.001 | 3.748 | 3.588 | 0.000 |  |  |  |  |
| Frontal_Sup_2_R (aal3v1) |  | 14 | 30 | 44 | 0.069 | 0.000 | 4.643 | 4.359 | 0.000 |  | 0.005 | 707 | 0.001 |
| Cingulate_Mid_R (aal3v1) |  | 10 | 44 | 30 | 0.081 | 0.000 | 4.589 | 4.314 | 0.000 |  |  |  |  |
| Right Cingulate Gyrus |  | 18 | 20 | 34 | 0.652 | 0.001 | 3.737 | 3.578 | 0.000 |  |  |  |  |
| Parietal_Sup_R (aal3v1) |  | 18 | -66 | 50 | 0.082 | 0.000 | 4.587 | 4.312 | 0.000 |  | 0.092 | 288 | 0.024 |
| Right Precuneus |  | 16 | -54 | 44 | 0.132 | 0.000 | 4.429 | 4.178 | 0.000 |  |  |  |  |
| Cingulate_Mid_L (aal3v1) |  | -6 | -28 | 36 | 0.365 | 0.001 | 4.039 | 3.844 | 0.000 |  | 0.596 | 73 | 0.224 |
| Cingulate_Mid_R (aal3v1) |  | 10 | -30 | 34 | 0.446 | 0.001 | 3.947 | 3.764 | 0.000 |  | 0.695 | 54 | 0.295 |
| Frontal_Inf_Oper_R (aal3v1) |  | 30 | 8 | 34 | 0.466 | 0.001 | 3.926 | 3.745 | 0.000 |  | 0.460 | 103 | 0.153 |
| Frontal_Mid_2_R (aal3v1) |  | 38 | 16 | 38 | 0.654 | 0.001 | 3.735 | 3.577 | 0.000 |  |  |  |  |
| Frontal_Mid_2_R (aal3v1) | 9 | 36 | 32 | 34 | 0.521 | 0.001 | 3.869 | 3.695 | 0.000 |  | 0.701 | 53 | 0.299 |
| Cingulate_Mid_L (aal3v1) | 32 | -2 | 12 | 40 | 0.864 | 0.002 | 3.490 | 3.358 | 0.000 |  | 0.818 | 32 | 0.422 |
| Calcarine_R (aal3v1) | 18 | 18 | -86 | 16 | 0.893 | 0.002 | 3.444 | 3.317 | 0.000 |  | 0.795 | 36 | 0.394 |
| Cuneus_R (aal3v1) |  | 14 | -80 | 22 | 0.929 | 0.003 | 3.374 | 3.254 | 0.001 |  |  |  |  |
| Temporal_Inf_R (aal3v1) |  | 38 | 6 | -42 | 0.000 | 0.000 | -7.042 | 6.201 | 0.000 |  | 0.000 | 1515 | 0.000 |
| Temporal_Pole_Mid_R (aal3v1) |  | 42 | 16 | -32 | 0.000 | 0.000 | -6.610 | 5.894 | 0.000 |  |  |  |  |
| Temporal_Inf_R (aal3v1) | 20 | 46 | -4 | -40 | 0.001 | 0.000 | -5.952 | 5.405 | 0.000 |  |  |  |  |
| Insula_R (aal3v1) |  | 40 | -8 | -6 | 0.235 | 0.008 | -4.219 | 3.999 | 0.000 |  | 0.690 | 55 | 0.290 |
| Left NTLE vs Control | | | | | | | | | | | | | |
| Right Superior Temporal Gyrus |  | 38 | -32 | 8 | 0.140 | 0.134 | 4.410 | 4.113 | 0.000 |  | 0.159 | 245 | 0.051 |
| Right Extra-Nuclear |  | 38 | -28 | -2 | 0.339 | 0.134 | 4.059 | 3.821 | 0.000 |  |  |  |  |
| Left Extra-Nuclear |  | -22 | -6 | 18 | 0.274 | 0.134 | 4.150 | 3.897 | 0.000 |  | 0.213 | 206 | 0.071 |
| Left Extra-Nuclear |  | -22 | -18 | 16 | 0.505 | 0.134 | 3.863 | 3.654 | 0.000 |  |  |  |  |
| Thal_VPL_L (aal3v1) |  | -20 | -22 | 6 | 0.680 | 0.134 | 3.677 | 3.494 | 0.000 |  |  |  |  |
| Left Frontal Lobe |  | -18 | -22 | 46 | 0.430 | 0.134 | 3.947 | 3.726 | 0.000 |  | 0.526 | 89 | 0.220 |
| Supp_Motor_Area_R (aal3v1) |  | 10 | -24 | 56 | 0.433 | 0.134 | 3.944 | 3.723 | 0.000 |  | 0.768 | 37 | 0.430 |
| Right Cerebellum Anterior Lobe |  | 14 | -48 | -30 | 0.468 | 0.134 | 3.904 | 3.689 | 0.000 |  | 0.737 | 43 | 0.394 |
| Right Cerebellum Anterior Lobe |  | 24 | -52 | -34 | 0.921 | 0.134 | 3.348 | 3.206 | 0.001 |  |  |  |  |
| Right Frontal Lobe |  | 20 | -18 | 46 | 0.476 | 0.134 | 3.895 | 3.682 | 0.000 |  | 0.757 | 39 | 0.418 |
| Right Extra-Nuclear |  | 28 | -12 | 18 | 0.635 | 0.134 | 3.725 | 3.536 | 0.000 |  | 0.222 | 201 | 0.074 |
| Right Extra-Nuclear |  | 22 | -18 | 2 | 0.794 | 0.134 | 3.546 | 3.380 | 0.000 |  |  |  |  |
| Right Extra-Nuclear |  | 24 | 6 | 18 | 0.854 | 0.134 | 3.464 | 3.308 | 0.000 |  |  |  |  |
| Right Medial Frontal Gyrus |  | 14 | 34 | -10 | 0.724 | 0.134 | 3.629 | 3.453 | 0.000 |  | 0.727 | 45 | 0.383 |
| Right Frontal Lobe |  | 22 | 30 | -6 | 0.931 | 0.134 | 3.324 | 3.184 | 0.001 |  |  |  |  |
| Right Middle Occipital Gyrus |  | 30 | -72 | 6 | 0.780 | 0.134 | 3.564 | 3.396 | 0.000 |  | 0.757 | 39 | 0.418 |
| Calcarine_R (aal3v1) |  | 22 | -76 | 4 | 0.938 | 0.134 | 3.308 | 3.170 | 0.001 |  |  |  |  |
| Cerebellum_4_5_R (aal3v1) |  | 12 | -46 | -4 | 0.875 | 0.134 | 3.433 | 3.281 | 0.001 |  | 0.804 | 30 | 0.480 |
| Temporal_Mid_L (aal3v1) |  | -62 | -36 | -10 | 0.004 | 0.007 | -5.571 | 5.025 | 0.000 |  | 0.014 | 615 | 0.004 |
| Temporal_Mid_L (aal3v1) | 21 | -62 | -20 | -6 | 0.004 | 0.007 | -5.564 | 5.020 | 0.000 |  |  |  |  |
| Temporal_Mid_L (aal3v1) |  | -60 | -20 | -22 | 0.057 | 0.012 | -4.721 | 4.366 | 0.000 |  |  |  |  |
| Angular_L (aal3v1) |  | -52 | -60 | 28 | 0.062 | 0.013 | -4.695 | 4.345 | 0.000 |  | 0.195 | 218 | 0.064 |
| Temporal_Mid_L (aal3v1) |  | -54 | -56 | 16 | 0.631 | 0.061 | -3.730 | 3.540 | 0.000 |  |  |  |  |
| Parietal_Inf_L (aal3v1) |  | -32 | -60 | 50 | 0.367 | 0.039 | -4.022 | 3.789 | 0.000 |  | 0.288 | 167 | 0.100 |
| Right NTLE vs Control | | | | | | | | | | | | | |
| Cerebellum_8_L (aal3v1) |  | -34 | -42 | -44 | 0.737 | 0.199 | 3.686 | 3.473 | 0.000 |  | 0.320 | 149 | 0.104 |
| Cerebellum_6_L (aal3v1) |  | -36 | -44 | -28 | 0.901 | 0.199 | 3.455 | 3.275 | 0.001 |  |  |  |  |
| Cerebellum_4_5_L (aal3v1) |  | -30 | -36 | -30 | 0.913 | 0.199 | 3.431 | 3.255 | 0.001 |  |  |  |  |
| Left Medial Frontal Gyrus |  | -18 | 52 | -2 | 0.796 | 0.199 | 3.615 | 3.412 | 0.000 |  | 0.824 | 29 | 0.468 |
| Cerebellum_6_L (aal3v1) |  | -12 | -66 | -12 | 0.799 | 0.199 | 3.610 | 3.408 | 0.000 |  | 0.144 | 247 | 0.042 |
| Cerebellum_4_5_L (aal3v1) |  | -6 | -46 | 0 | 0.877 | 0.199 | 3.497 | 3.311 | 0.000 |  |  |  |  |
| Cerebellum_6_L (aal3v1) |  | -14 | -74 | -16 | 0.925 | 0.199 | 3.406 | 3.233 | 0.001 |  |  |  |  |
| Thal_LGN_L (aal3v1) |  | -22 | -24 | -4 | 0.840 | 0.199 | 3.554 | 3.361 | 0.000 |  | 0.802 | 33 | 0.437 |
| Temporal_Mid_R (aal3v1) |  | 64 | -32 | -2 | 0.010 | 0.016 | -5.414 | 4.834 | 0.000 |  | 0.000 | 1812 | 0.000 |
| Temporal_Mid_R (aal3v1) | 21 | 62 | -46 | 0 | 0.042 | 0.016 | -4.932 | 4.475 | 0.000 |  |  |  |  |
| Temporal_Inf_R (aal3v1) |  | 60 | -20 | -26 | 0.045 | 0.016 | -4.913 | 4.461 | 0.000 |  |  |  |  |
| Precentral_R (aal3v1) |  | 54 | -10 | 40 | 0.442 | 0.026 | -4.014 | 3.746 | 0.000 |  | 0.509 | 93 | 0.192 |
| Postcentral_R (aal3v1) |  | 60 | -14 | 36 | 0.772 | 0.044 | -3.645 | 3.438 | 0.000 |  |  |  |  |
| Precentral_R (aal3v1) |  | 48 | 6 | 44 | 0.661 | 0.036 | -3.772 | 3.545 | 0.000 |  | 0.792 | 35 | 0.423 |
| Frontal_Sup_2_R (aal3v1) |  | 22 | -6 | 70 | 0.738 | 0.041 | -3.686 | 3.472 | 0.000 |  | 0.861 | 22 | 0.532 |

K_E_, cluster size; FDR, false discovery rate; FWE, family wise error; MTLE, mesial temporal lobe epilepsy; NTLE, neocortical temporal lobe epilepsy.
